# Reversal of Surfactant Protein B Deficiency in Patient Specific Human Induced Pluripotent Stem Cell Derived Lung Organoids by Gene Therapy

**DOI:** 10.1038/s41598-019-49696-8

**Published:** 2019-09-17

**Authors:** Sandra Lawrynowicz Leibel, Alicia Winquist, Irene Tseu, Jinxia Wang, Daochun Luo, Sharareh Shojaie, Neal Nathan, Evan Snyder, Martin Post

**Affiliations:** 1 Department of Pediatrics, University of California, San Diego, Rady Children’s Hospital, San Diego, La Jolla, CA USA; 2 Sanford Burnham Prebys Medical Discovery Institute, La Jolla, CA USA; 3 Sanford Consortium for Regenerative Medicine, La Jolla, CA USA; 4 Translational Medicine Program, Peter Gilgan Centre for Research and Learning, Hospital for Sick Children, Toronto, Ontario Canada; 5 Department of Physiology, University of Toronto, Toronto, Ontario Canada

**Keywords:** Organogenesis, Respiratory tract diseases, Pluripotent stem cells

## Abstract

Surfactant protein B (SFTPB) deficiency is a fatal disease affecting newborn infants. Surfactant is produced by alveolar type II cells which can be differentiated *in vitro* from patient specific induced pluripotent stem cell (iPSC)-derived lung organoids. Here we show the differentiation of patient specific iPSCs derived from a patient with SFTPB deficiency into lung organoids with mesenchymal and epithelial cell populations from both the proximal and distal portions of the human lung. We alter the deficiency by infecting the SFTPB deficient iPSCs with a lentivirus carrying the wild type SFTPB gene. After differentiating the mutant and corrected cells into lung organoids, we show expression of SFTPB mRNA during endodermal and organoid differentiation but the protein product only after organoid differentiation. We also show the presence of normal lamellar bodies and the secretion of surfactant into the cell culture medium in the organoids of lentiviral infected cells. These findings suggest that a lethal lung disease can be targeted and corrected in a human lung organoid model *in vitro*.

## Introduction

Lung development is a complicated process that involves the specialization of multiple bronchiolar and alveolar epithelial populations. The primordial lung bud expresses Nkx2 homeobox 1 (NKX2-1), an important transcription factor in lung development^1^ that also activates surfactant related genes^2^. Alveolar type II (ATII) epithelial cells synthesize, secrete and recycle all components of surfactant and dysfunction in surfactant metabolism can result in a variety of pediatric lung diseases including respiratory distress syndrome and interstitial lung disease^3^. To understand the various mechanisms driving disease, it is important to have a good model system *in vitro* that can recapitulate the *in vivo* disease process.

Lung organoids have been successfully used to study lung development and disease^4–8^. They are 3-dimensional, complex, multicellular structures that can be derived from patient specific human induced pluripotent stem cells (hiPSCs). hiPSC derived lung organoids represent early stages (pseudoglandular and canalicular) of lung development^9,10^ and can contain multicellular structures^9,11^ or be pure alveolar spheroids^12,13^. They can be used to study cellular and metabolic biology without the use of an animal model or fetal tissue. Although many differentiation protocols in the literature have been successful in mimicking lung development from stem cells, there has not been an examination of how a specific mutation impacts the differentiation process including its effects on the early endoderm, as well as the proximal and distal lung epithelial cell populations in the lung organoids.

Inherited deficiency of surfactant protein B (SFTPB) is a rare genetic cause of lethal respiratory distress syndrome in full-term newborn infants^14,15^. It is most commonly caused by a homozygous, frameshift, loss of function mutation of the surfactant protein B gene (p.Pro133GlnfsTer95, previously known as 121ins2)^16–18^ making it a good target for gene therapy. Gene therapy has been utilized successfully to repair or inactivate mutations in animal models of monogenic human diseases^19^ as well as human *in vitro* cells^12^. There have been successful attempts at gene editing SFTPB deficiency including nuclease encoding mRNA, electroporation-mediated gene delivery and CRISPR^12,19–21^, but these methods have a low efficiency of mutation correction and are time intensive. Lentiviral (LV) vectors of the Retroviridae family show interesting properties for monogenic gene therapy, since they integrate into the host genome and allow long-lasting gene expression^22^. Lentiviral correction of monogenic mutations has been used in human disease^22,23^, is a highly efficient process and can be used to induce overexpression of proteins of interest after intratracheal delivery in animal models^24^. Infection of iPSCs with lentiviral inserts is a highly efficient process since stem cells grow quickly, remain undifferentiated in specific cell culture conditions and can establish fully infected clones within 2–3 passages. Lentiviral infection has also been targeted in lung epithelial cells^24^ but these grow much slower *in vitro* and are prone to dedifferentiation.

In this study we generate SFTPB-deficient (p.Pro133GlnfsTer95) hiPSCs from patient specific fibroblasts and use a modified differentiation technique to transform them into 3-dimensional (3D) human lung organoids containing both epithelial and mesenchymal cell populations of the proximal and distal airways to closely replicate the human fetal lung. We employ highly efficient lentiviral infection of the wild type SFTPB gene into the mutated hiPSC line and show successful transcription and translation of SFTPB at the organoid level, the presence of lamellar bodies in ATII cells as well as the secretion of surfactant bioactive lipids by functional ATII cells.

## Results

### Generation of patient specific iPS cells containing the SFTPB deficiency

With parental consent, a skin biopsy of a patient with the p.Pro133GlnfsTer95 (hereafter known as Pro133) SFTPB mutation^18^ was obtained and expanded in culture. The fibroblasts were then reprogrammed into induced pluripotent stem cells (iPSCs) using a highly efficient Sendai-virus (RNA virus) based system^25^ carrying the transcription factors hOct4, hSox2, hKlf4 and hMyc. The resulting hiPSCs (hiPro133 cells) expressed the pluripotency markers OCT4, NANOG, TRA-1-60 and SSEA4 (Supplementary Fig. S1) and the karyotype was normal (Supplementary Fig. S2). Genomic sequencing of the hiPSCs confirmed the presence of the frameshift mutation caused by the substitution of GAA for the nucleotide C (Supplementary Fig. S2). The Pro133 mutation introduces a Sfu I restriction enzyme site that permits detection of the mutation with restriction enzyme digestion^26^. The homozygous mutation was confirmed with Sfu1 digestion revealing two distinct bands (210 and 103 bp) in the SFTPB deficient iPSCs and fibroblasts, one band (311 bp) in the wild type fibroblasts and three bands in the heterozygous fibroblasts (Supplementary Fig. S2).

### Infection of hPro133 iPS cells with a lentivirus carrying the SFTPB cDNA sequence

**Figure 1:**
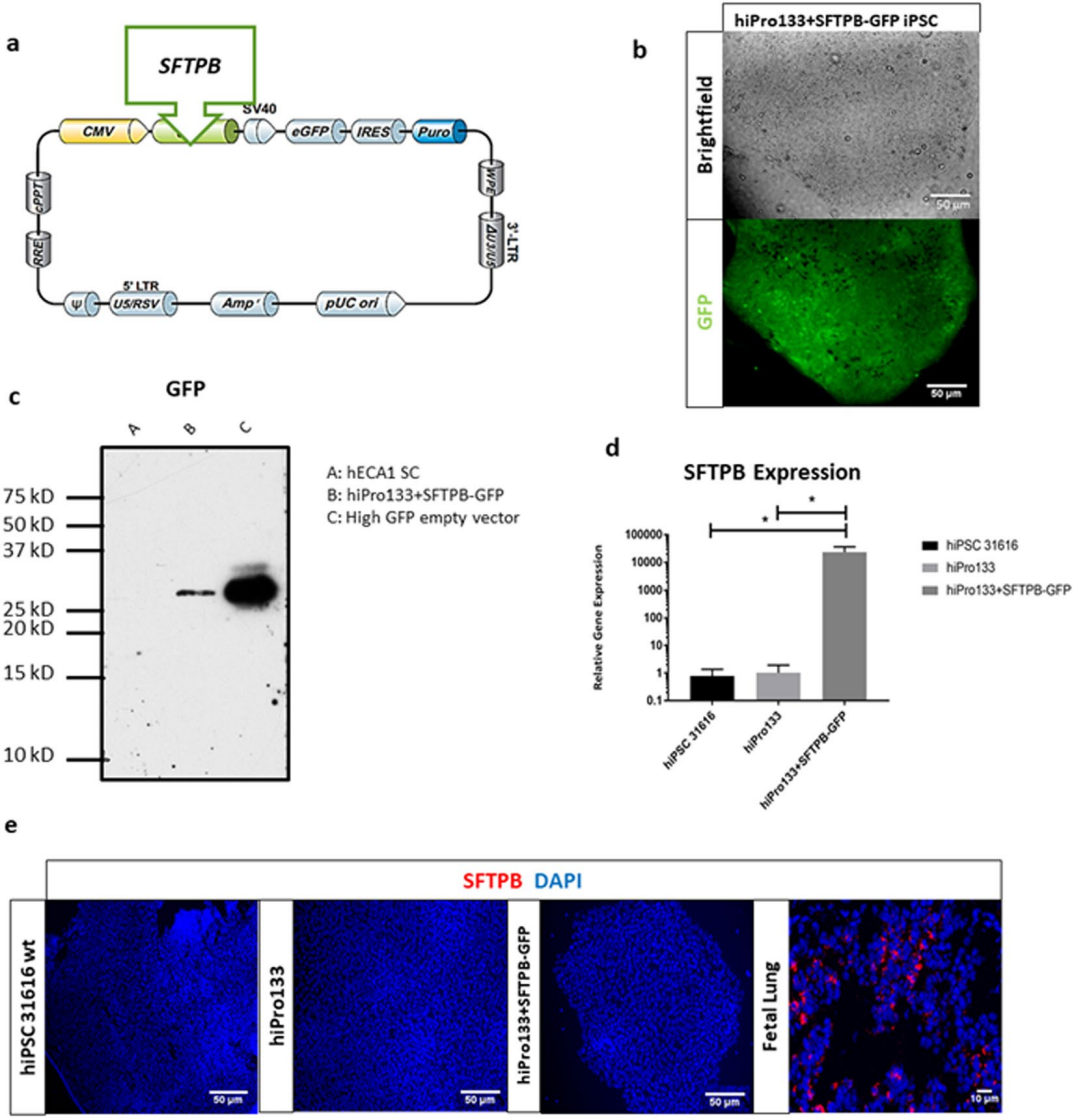
Human hiPro133 iPS cells were successfully infected with a lentivirus carrying the pre-form SFTPB cDNA sequence. (**a**) A schematic of the lentiviral vector containing a CMV promoter driving SFTPB followed by SV40-eGFP-IRES-puromycin for selection. The lentiviral infected iPSCs hereafter known as hiPro133 + SFTPB-GFP (**b**) A phase contrast (top) and GFP live cell (bottom) image of hiPro133 + SFTPB-GFP cells (Scale bar = 50 μ*m*). (**c**) Western blot of GFP expression comparing a wild type non-infected heCA1 stem cell, the hiPro133 + SFTPB-GFP cell and an empty vector control (exposure time 2 minutes). (**d**) Gene expression of SFTPB in the hiPro133 + SFTPB-GFP cell line compared to an untransduced hiPro133 and wt 31616 iPS cell lines. * represents significant p-value <0.05 (n = 3 iPSC colonies). (**e**) Immunofluorescence examining protein expression of SFTPB in the iPSC cell lines hi31616, hiPro133 and hiPro133 + SFTPB-GFP and fetal lung tissue. No expression was detected on 3 different staining procedures compared to the positive control. (Scale bar = 50 μ*m*).

We used a commercially available lentiviral vector construct containing a CMV promoter which constitutively drove the open reading frame of homo sapiens SFTPB gene (Fig. 1a). This was followed by a SV40 promoter driving eGFP-IRES-puromycin to aid with selection after infection. We observed GFP-positive iPSC colonies that were successfully infected by the lentiviral vector (hereafter called hiPro133 + SFTPB-GFP) (Fig. 1b) and the GFP protein was confirmed through immunoblotting of the transduced cells (Fig. 1c). We also measured SFTPB gene expression in the transduced iPS cells in comparison to the SFTPB-deficient iPS cells (hiPro133) and a normal control iPSC line, 31616 (Fig. 1d). Expression levels of SFTPB were measured by qPCR and normalized to the housekeeping gene ACTB (β-actin). SFTPB mRNA expression was significantly higher in the hiPro133 + SFTPB-GFP iPSCs compared to hiPro133 and hi31616 cells. Finally, we examined whether the translation of the SFTPB gene product occurred at the iPSC level in the same cell lines through immunofluorescence using a SFTPB antibody that detects both the pro- and mature forms of SFTPB. As compared to our positive control human fetal lung, there was no protein expression of SFTPB in the hi31616, hiPro133 and hiPro133 + SFTPB-GFP iPSC lines (Fig. 1e).

### Differentiation of hPro133 + SFTPB-GFP and hPro133 iPS cells into lung progenitor cells

**Figure 2:**
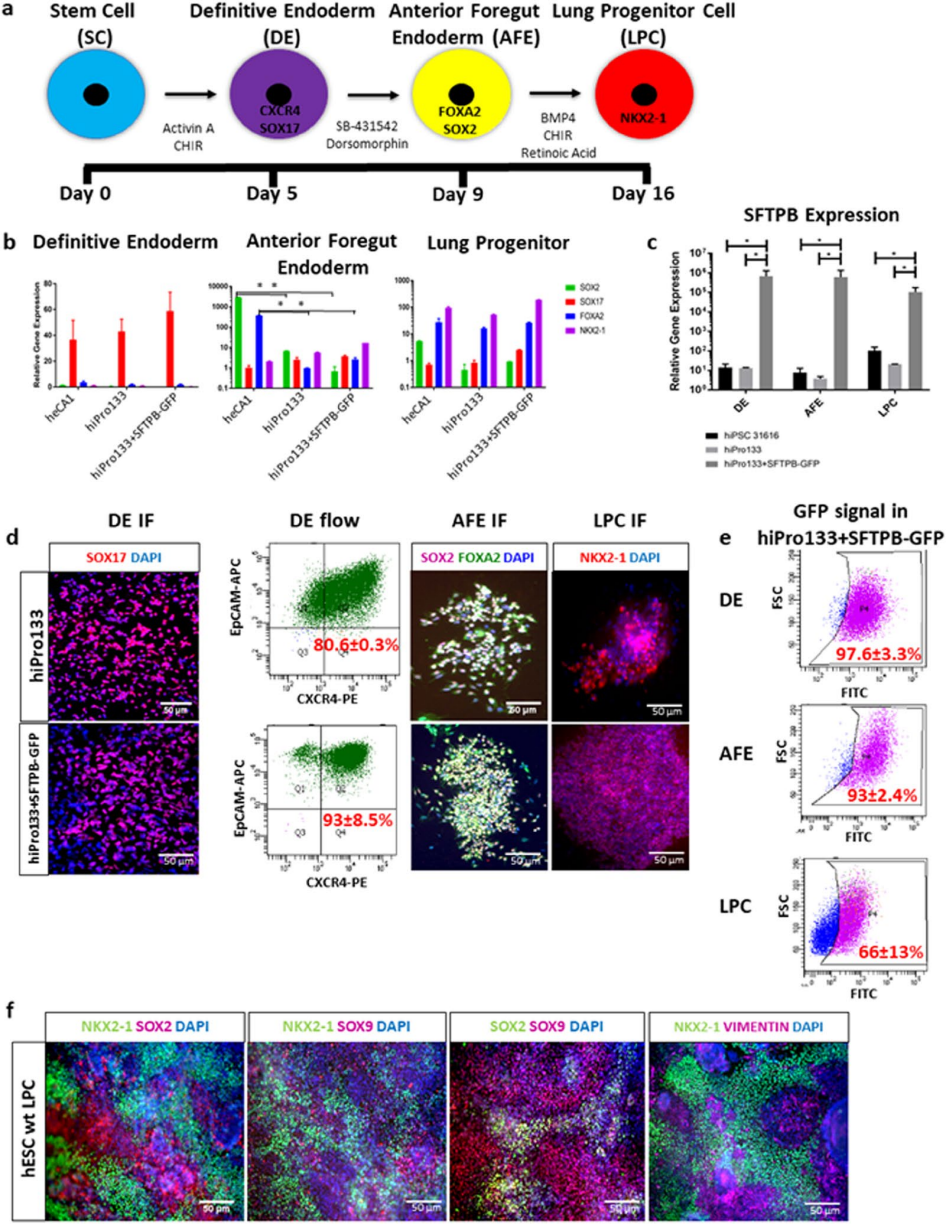
Endodermal differentiation protocol and results of human CA1 ESCs, and 31616 wt, hiPro133 and hiPro133 + SFTPB-GFP iPS cells to lung progenitor cells. (**a**) Timeline showing culture steps from embryonic stem/human pluripotent stem cells to definitive endoderm (DE), anterior foregut endoderm (AFE) and lung progenitor (LPC) cells, including the markers expressed by the desired cell type and the combination of growth factors and small molecules used for the differentiation. (**b**) Gene expression profiles of common markers of endodermal differentiation of the wt heCA1, hiPro133 and hiPro133 + SFTPB-GFP cells at DE, AFE, and LPC. * represents significant p-value < 0.05. (n = 3–5 separate differentiations). (**c**) Gene expression of SFTPB during the differentiation of wt hi31616, mutant hiPro133, and rescued hiPro133 + SFTPB-GFP cells at DE, AFE and LPC. * represents significant p-value < 0.05. (n = 3 separate differentiations). (**d**) Immunofluorescence for DE marker SOX17, AFE markers FOXA2 and SOX2 and LPC marker NKX2-1 in hiPro133 and hiPro133 + SFTPB-GFP differentiated cells. (n = 3–5 separate differentiations; Scale bar = 50 μ*m*). Second column shows flow cytometry results for definitive endoderm markers CXCR4 and EPCAM of hiPro133 and hiPro133 + SFTPB-GFP cells. Double positive CXCR4/EPCAM cells represent DE cell differentiation efficiency as a mean ± SEM. Representative of > 4 experiments. (**e**) Scatter plot from a flow representing the endogenous GFP expression of hiPro133 + SFTPB-GFP cells at DE, AFE and LPC from 3 separate differentiations, represented as mean ± SEM. Unstained hiPro133 DE cells served as negative control. (**f**) Immunofluorescence of wt hESC LPC monolayers for NKX2-1, SOX2, SOX9 and vimentin. DAPI is represented in blue. Fluorophores of antibodies as labelled on image. (n = 4 differentiations; Scale bar = 50 μ*m*).

The mutant, wild type and corrected iPSC clones underwent successful endodermal differentiation into NKX2-1 expressing lung progenitor cells^27,28^. Briefly, mimicking fetal lung development, monolayer cultures were exposed to a cocktail of growth factors and small molecules to become definitive endoderm (DE)^29^, anterior foregut endoderm (AFE)^30^ and lung progenitor cells (LPC)^31,32^ (Fig. 2a). Throughout the differentiation process, gene expression analyses of the human embryonic stem cell line CA1 (heCA1) and/or the iPSC line hi31616 served as normative controls. Relative gene expression throughout endodermal differentiation was analyzed using qPCR normalized to ACTB. Gene expression profiles during the differentiation from stem cell to lung progenitor cell between the heCA1, hiPro133 and hiPro133 + SFTPB-GFP cell lines, showed no difference during DE and LPC induction, but in AFE the expression of SOX2 and FOXA2 was statistically greater in the heCA1 cell line compared to the hiPro133 and hiPro133 + SFTPB-GFP cell lines (Fig. 2b).

To ensure robustness of transgene expression in the hiPro133 + SFTPB-GFP cell line, the cells were exposed to puromycin at 0.25 ug/mL for 7 days prior to differentiation. GFP expression was checked by FACS at each stage and a robust signal of >95% FITC (GFP) positive cells was consistently detected in the hiPro133 + SFTPB-GFP cell line through DE and AFE with a slight decrease in signal noted in LPC (Fig. 2e). In conjunction with the GFP signal, gene expression of SFTPB was analyzed throughout the differentiation from hiPSC to LPC. Independent of the state of differentiation, SFTPB mRNA expression remained high in the hiPro133 + SFTPB-GFP cell line compared to the wild type hi31616 and mutant hiPro133 lines in line with the constitutive nature of the CMV promoter in the hiPro133 + SFTPB-GFP cell line (Fig. 2c).

Differentiation efficiency for each cell line at each stage of endodermal differentiation was evaluated. After exposure to elevated levels of human Activin A for 3–4 days, the hiPSC derived DE cells expressed the cell surface markers CXCR4 and EPCAM through fluorescence-activated cell sorting (FACS) and SOX17 through immunofluorescence (Fig. 2d). Our differentiations consistently yielded an 80–95% pure population of hiPro133 and hiPro133 + SFTPB-GFP iPSC derived DE cells. Induction into AFE cells was confirmed by the co-expression of FOXA2 and SOX2^33^ using immunofluorescence (Fig. 2d) with an efficiency of 70–90% in hiPro133 and hiPro133 + SFTPB-GFP iPSC derived AFE cells. Finally, LPC induction was confirmed with immunofluorescence staining using an antibody against the transcription factor NKX2-1 (Fig. 2d) with an efficiency of 30–50% in hiPro133 and hiPro133 + SFTPB-GFP iPSC derived LPC cells.

At the end of endodermal differentiation, prior to passing into 3D Matrigel, the LPCs were evaluated through immunostaining for NKX2-1, SOX2, SOX9 and vimentin. SOX2 is a marker of early lung and esophageal development and is expressed in the dorsal endoderm with NKX2-1 expression restricted to the ventral endoderm^34^. SOX9 expression is restricted to the cartilage of the trachea and progenitor cells important for branching morphogenesis in early lung development^35,36^. Vimentin is a marker for mesenchymal cells that are important in early lung development. Mesenchyme is derived from the mesodermal germ layer and is an indicator of the purity of the endodermal differentiation. The antibodies to the markers described above revealed distinct populations of epithelial (NKX2-1^+^) and mesenchymal (VIM^+^) cells at the lung progenitor stage (Fig. 2f). We noted that an efficient differentiation contained less VIM^+^ cells (36 ± 4%) while a non-efficient differentiation contained more VIM^+^ cells (64 ± 8%) (mean ± s.e.m., n = 3 independent differentiations). Many investigators sort out the NKX2-1 positive cells using cell surface antigens in order to purify the endodermal population prior to passage into 3D Matrigel to make lung organoids^27,32^. We were interested in modeling lung development with a combination of endodermal and mesodermal elements to replicate epithelial-mesenchymal cross-talk and, therefore, passed the VIM^+^ cells along with the NKX2-1^+^ cells into 3D Matrigel from efficient LPC differentiations (NKX2-1^+^ >49 ± 14%). We also discovered the presence of single populations of SOX2^+^ (31 ± 4%), SOX9^+^ (31 ± 1%), and NKX2-1^+^ (49 ± 14%) cells and occasionally double positive SOX2/SOX9 cells (15 ± 8.9%) in hESC LPCs (mean ± s.e.m., n > 4 independent differentiations) (Fig. 2f).

### Generation of 3D lung organoids from human hESC wt, hiPro133 + SFTPB-GFP and hiPro133 lung progenitor cells

**Figure 3:**
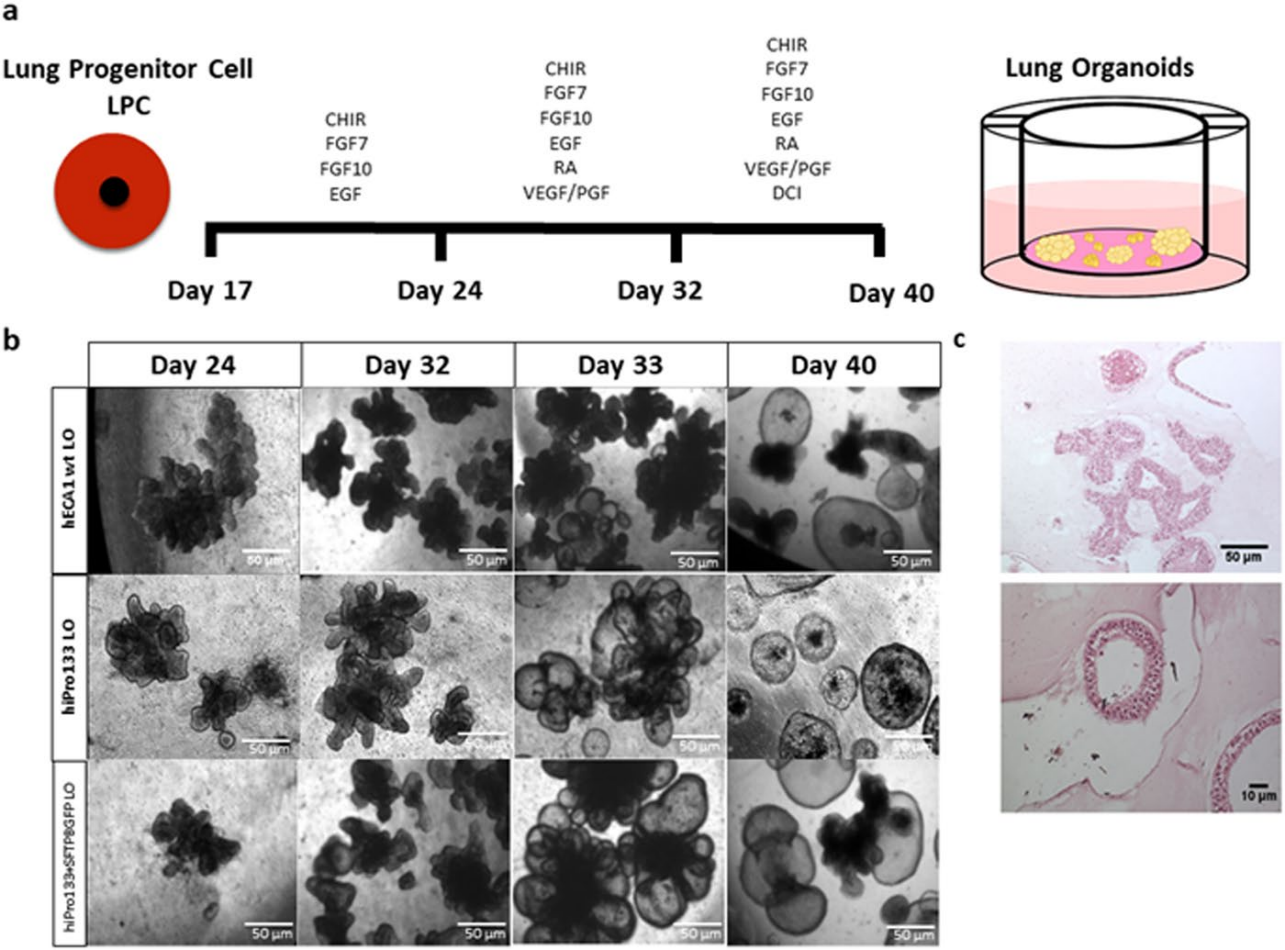
Timeline and histology of the differentiation of human ES/iPS-derived lung progenitor cells to 3D lung organoids. (**a**) Timeline showing monolayer culture LPCs passed to 3D Matrigel in transwell inserts with corresponding growth factors and small molecules and days of differentiation. (**b**) Representative live phase contrast images of developing 3D lung organoids at different time points from wt heCA1, hiPro133 and hiPro133 + SFTPB-GFP cells. (Scale bar = 50 μ*m*). (**c**) Hematoxylin and eosin images of wt heCA1 day 40 lung organoids. (Scale bar = 50 μ*m*).

For the induction of 3D lung organoids, we passed the LPC monolayer as small aggregates containing both endodermal and mesodermal cells. The timeline for passaging LPC cells into matrigel-containing transwell inserts, along with the addition of growth factors and small molecules at specific time points during the differentiation is shown in Fig. 3a. Live phase contrast images of hESC wt, hiPro133 and hiPro133 + SFTPB-GFP LPC aggregates as they differentiate into lung organoids are shown in Fig. 3b. For our 3D lung organoid differentiation, we used chemical signals important in embryonic lung development to differentiate the LPCs into 3D lung organoids^37–40^. After passaging into 3D matrigel, the growth factors FGF7, FGF10 and EGF were used due to their importance in branching morphogenesis, growth and differentiation^41,42^. Since Wnt signaling is also important the glycogen synthase kinase (GSK-3) inhibitor CHIR was also added^43^. In the second week, RA and VEGF/PGF were added due to their importance in alveolar cell formation^44,45^. Finally, for epithelial maturation, a cocktail of dexamethasone, cAMP and isobutylxanthine was added in the final week^40,46^. Morphologically, one to two weeks after passaging LPC aggregates into 3D matrigel, there were multiple 3D organoids of mainly branching morphologies, but once DCI was added, the branching organoids were altered into more spherical forms by day 40 (Fig. 3b). At day 40, H&E staining was performed on the organoids which showed multiple cellular types including columnar and squamous epithelium (Fig. 3c).

### Lung organoids from human hESC, hiPro133 and hiPro133 + SFTPB-GFP cells express branching tip and airway markers

**Figure 4:**
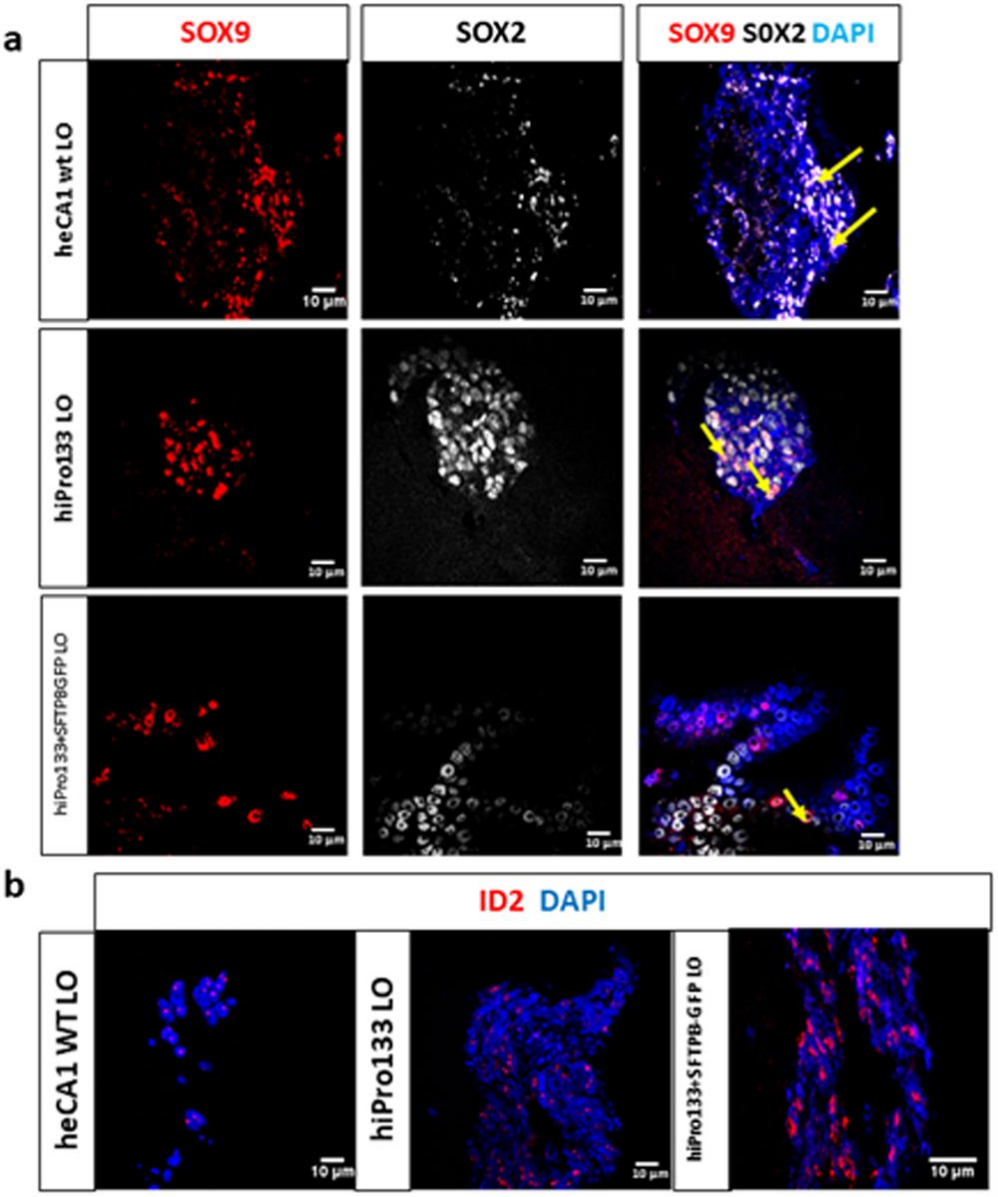
Branching tip and airway markers in lung organoids derived from wt heCA1, hiPro133 and hiPro133 + SFTPB-GFP stem cells. (**a**) Immunofluorescent expression of SOX2 and SOX9 in day 40 lung organoids from wt heCA1, hiPro133 and hiPro133 + SFTPB-GFP cells. Yellow arrows indicate double positive SOX9/SOX2 cells. Representative of >6–15 independent experiments. (Scale bar = 10 μ*m*). (**b**) Immunofluorescent expression of ID2 in day 40 lung organoids from wt heCA1, hiPro133 and hiPro133 + SFTPB-GFP cells. Representative of >6–15 independent experiments. (Scale bar = 10 μ*m*).

At the LPC stage, we identified epithelial cells positive for NKX2-1 and mesenchymal cells positive for vimentin (Fig. 2f). Therefore, by passaging them into 3D matrigel, we were expecting to more closely replicate fetal lung development and the epithelial-mesenchymal interactions important to branching morphogenesis. Our organoids expressed airway transcription factors such as SOX2 (proximal and branching marker) and SOX9 (branching and distal marker) and the bipotential branching tip progenitor ID2^47,48^. Occasionally, co-expression of both transcription factors was observed in the organoids (heCA1 wt 14 ± 7%, hiPro133 3 ± 2% and hiPro133 + SFTPB-GFP 8 ± 5% (mean ± s.e.m., n = 4 independent differentiations) (Fig. 4a). The organoids were also positive for the transcription factor ID2, a specific marker found at the tips of the lung during branching morphogenesis (Fig. 4b). The ID2 staining is consistent with previous publications^49^.

### Lung organoids from human hESC, hiPro133 and hiPro133 + SFTPB-GFP cells express epithelial and mesenchymal markers of the proximal and distal lung

**Figure 5:**
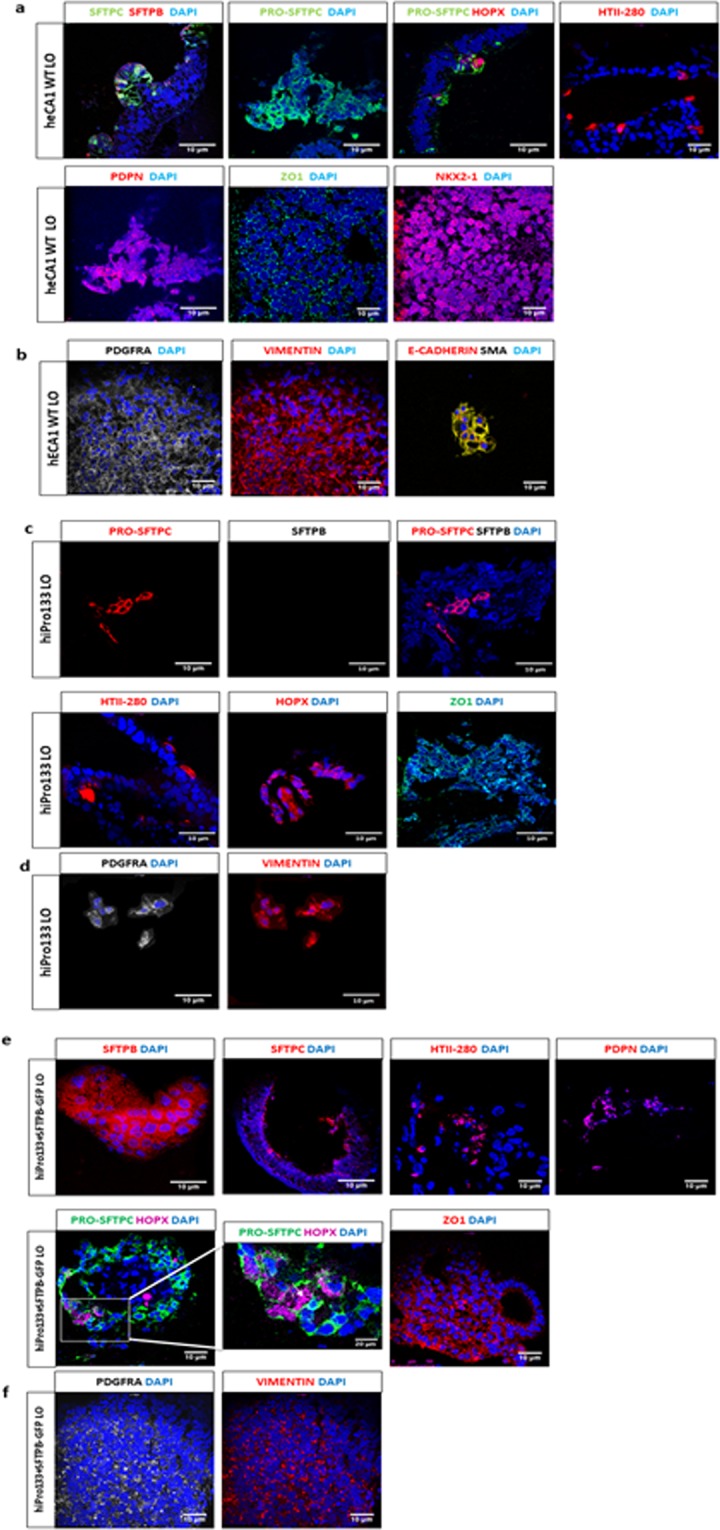
Expression of distal epithelial and mesenchymal lung markers in day 40 lung organoids derived from heCA1 cells, hiPro133 and hiPro133 + SFTPB-GFP stem cells. (**a**) Immunofluorescent expression of distal lung cell markers SFTPC, SFTPB, PRO-SFTPC, HOPX, HTII-280, PDPN, ZO-1 and NKX2-1 in day 40 wt heCA1 lung organoids. White arrow indicates the double positive HOPX/PRO-SFTPC cells. Data are representative of >15 independent experiments. (Scale bar = 10 μ*m*). (**b**) Immunofluorescent expression of mesenchymal markers PDGFRA and vimentin in day 40 wt heCA1 lung organoids. Data are representative of >15 independent experiments. (Scale bar = 10 μ*m*). (**c**) Immunofluorescent expression of distal lung cell markers PRO-SFTPC, SFTPB, HTII-280, HOPX, and ZO-1 in day 40 hiPro133 lung organoids. Data are representative of >15 independent experiments. Scale bars 10 µM (**d**) Expression of mesenchymal markers PDGFRA and vimentin in day 40 hiPro133 lung organoids. Representative of >6 independent experiments. Scale bars 10 uM. (**e**) Immunofluorescent expression of distal lung cell markers SFTPB, SFTPC, HTII-280, PDPN, PRO-SPC, HOPX, and ZO1 in day 40 hiPro133 + SFTPB-GFP lung organoids. White box represents area that is highlighted. White arrows indicate the double positive HOPX/PRO-SFTPC cells. Representative of >6 independent experiments. (Scale bar = 10 μ*m*). (**d**) Expression of mesenchymal markers PDGFRA and vimentin in day 40 hiPro133 + SFTPB-GFP lung organoids. Representative of >6 independent experiments. (Scale bar = 10 μ*m*).

After 6 weeks, lung organoids from heCA1 cells expressed a variety of immature and mature alveolar cell markers including cytosolic pro-SFTPC co-expressed with the bipotential alveolar progenitor transcription co-factor HOPX, podoplanin (PDPN an alveolar type I (ATI) marker), mature SFTPC and SFTPB (co-expressed ATII markers), and surface ATII marker HTII-280 (Fig. 5a). Of the total cells in the distal organoids, 22 ± 5.4% stained positive for SFTPC and 14.5 ± 4.3% SFTPB (mean ± s.e.m., n = 4 independent differentiations). The transcription factor NKX2-1 that activates surfactant expression was abundantly expressed as was ZO-1 which is located on the intercellular tight junctions of alveolar cells (Fig. 5a). Furthermore, the organoids contained PDGFRA- and vimentin-positive mesenchymal cells and co-expressed the epithelial marker E-cadherin and the mesenchymal marker SMA (Fig. 5b).

We then applied our organoid differentiation protocol to the hiPro133 and hiPro133 + SFTPB-GFP iPS-derived lung progenitor cells. ATII-specific surfactant protein B (SFTPB) was not detected in the hiPro133 iPS-derived organoids but there was expression of HOPX and the tight junctional protein ZO-1 (Fig. 5c). There were a small number of cells in the organoids that expressed PRO-SFTPC (3.3 ± 1.3%, mean ± s.e.m., n = 3 independent differentiations) and HTII-280. PDGFRA- and vimentin-positive mesenchymal cells were also present in the hiPro133 organoids (Fig. 5d). In contrast, the hiPro133 + SFTPB-GFP iPSC-derived distal lung organoids abundantly expressed SFTPB and mature SFTPC (36 ± 8.6% and 18 ± 4.4% respectively, mean ± s.e.m., n = 3 independent experiments). The organoids also stained positively for the ATII cell marker HTII-280 and tight junctional protein ZO1. The bipotential distal progenitor cell marker HOPX was also expressed, sometimes in isolated HOPX^+^ cells or co-expressed with pro-SFTPC in the same cell^50^ (Fig. 5e). PDPN, an ATI cell marker, was also detected in the hiPro133 + SFTPB-GFP organoids. Like the control heCA1-derived lung organoids, PDGRFA^+^/VIM^+^ cells were present in the hiPro133 + SFTPB-GFP organoids (Fig. 5f).

The lung organoids derived from heCA1 wt, hiPro133, and hiPro133 + SFTPB-GFP cells also expressed gene transcripts from proximal airway epithelial cells such as SOX2, FOXJ1, KRT5, MUC5AC and SCGB1A1. Significant differences in KRT5 and FOXJ1 mRNA expression was noted between the wt and mutant cells (Supplementary Fig. S3). Independent of cell line, all lung organoids stained positively for FOXJ1, a transcription factor found in ciliated cells, as well as basal cell and possible lung stem cell markers P63 and KRT5, the secretory cell marker SCGB3A2 and airway epithelial cell marker MUC5AC (Supplementary Fig. S3).

### Alveolar type II cells within the lung organoids have functional lamellar bodies

**Figure 6:**
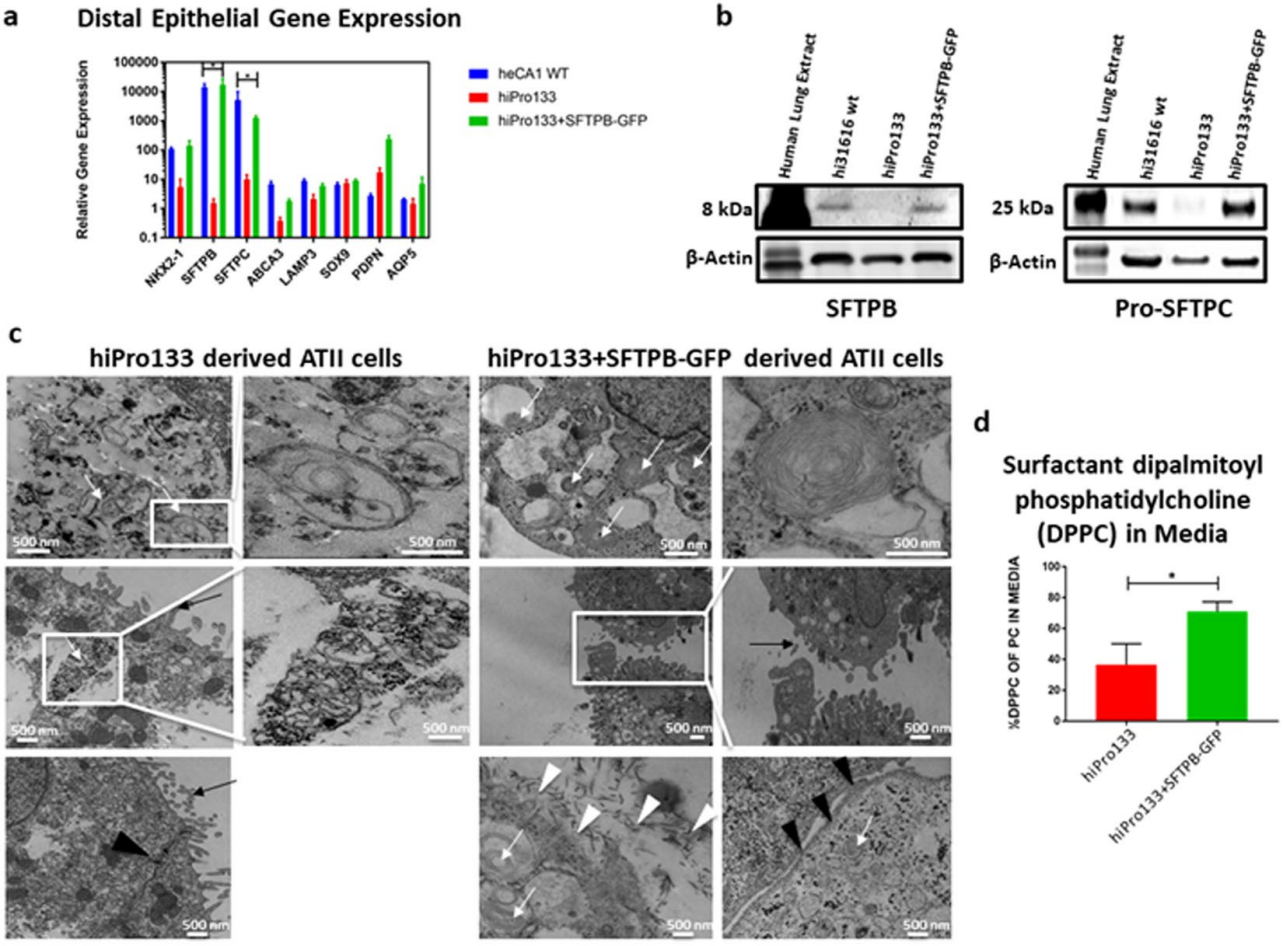
Expression and function of distal lung cells in stem cell derived day 40 wild type, hiPro133 and hiPro133 + SFTPB-GFP lung organoids. (**a**) Gene expression of alveolar epithelium and lamellar body related genes in day 40 heCA1 wt, hiPro133 and hiPro133 + SFTPB-GFP lung organoids (n = 3–5 separate differentiations). (**b**) Representative western blot of 8 kDa mature SFTBP and 25 kDa PRO-SFTPC expression in day 40 hi31616 wt, hiPro133 and hiPro133 + SFTPB-GFP lung organoids (n = 3 separate differentiations). (**c**) TEM images of day 40 hiPro133 and hiPro133 + SFTPB-GFP lung organoids. White arrows indicate lamellar bodies, black arrows indicate microvilli, white arrowheads indicate tubular myelin and black arrowheads indicate tight junctions. White boxes highlight the areas that have been magnified. (Scale bar = 500 nm). (**d**) Phosphatidylcholine mass spectrometric analysis of culture medium of hiPro133 and hiPro133 + SFTPB-GFP lung organoids (n = 3 independent differentiations, * represents significant p-value < 0.05).

Our human lung organoids derived from heCA1 and wild type and mutant hiPS cells expressed lamellar body specific transcripts SFTPB, SFTPC, ABCA3 and LAMP3 (Fig. 6a). However, expression of SFTPB and SFTPC were significantly down regulated in the hiPro133 organoids. Other lamellar body specific transcripts (ABCA3, LAMP3) were also decreased in the hiPro133 organoids. SFTPB is a highly processed protein which starts off as a 42 kD pre-pro-SFTPB product then gets processed into a 23–25 kDa pro-SFTPB and finally is present as the functional 8 kDa product in lamellar bodies^51^. Using Western blotting, we confirmed the presence of the functional 8 kDa SFTPB protein in the lung organoids derived from all cell lines except hiPro133 (Fig. 6b). The expression of PRO-SFTPC protein was also decreased in the hiPro133 lung organoids. The lamellar bodies of the ATII cells were examined using transmission electron microscopy. We detected ATII cells in the hiPro133 + SFTPB-GFP iPSC-derived organoids containing well-organized cytosolic lamellar bodies, microvilli at the apical cell membranes, tubular myelin external to the cells and tight junctions located between them (Fig. 6c). We also examined the lamellar bodies of the ATII cells in hiPro133 organoids. Consistent with published literature reports on infants affected with SFTPB deficiency^52,53^, we observed no mature lamellar bodies or tubular myelin although the ATII cells had intact microvilli and tight junctions (Fig. 6c). Pulmonary surfactant is made up of 10% protein and 90% lipids, with the most abundant surface-active lipid being dipalmitoyl phosphatidylcholine (DPPC)^54^. To assess the functionality of the ATII cells in the lung organoids, we analyzed the ability to secrete surfactant material into the cell culture medium. We analyzed the presence of surface-active DPPC in the medium of organoids from hiPro133 and hiPro133 + SFTPB-GFP cell lines using tandem mass spectrometry (MS/MS). The organoids that over-expressed SFTPB showed a significant increase in the percentage of surfactant-specific DPPC secreted into the medium (Fig. 6d). These MS/MS results suggest functionality of the ATII cells in the lung organoids.

## Discussion

Our data demonstrate successful genotypic and phenotypic duplication of a fatal SFTPB mutation *in vitro* using lung organoids. We developed a modified lung organoid differentiation protocol for a mutant iPS cell line derived from a patient with SFTPB deficiency (Pro133 iPS cells) and showed expression of progenitor, proximal and distal lung cell markers as well as lung mesenchyme. We confirmed the absence of SFTPB genetic and protein material in the ATII cells of these hiPro133 iPSC-derived lung organoids and showed abnormal lamellar body formation using TEM, consistent with both murine and human data.

We decided to focus on the expression of established marker proteins for lung progenitors and distal lung epithelial cells using flow, immunostaining and western blotting. Proteins are the final product of gene expression and, therefore, an excellent tool to track lung epithelial differentiation. However, in some instances we performed qPCR of genes of interest to confirm and strengthen the protein analyses.

We also successfully infected a lentivirus carrying the wild-type SFTPB coding region into patient specific SFTPB deficient (Pro133) iPS cells. We then differentiated the lentiviral infected (hiPro133 + SFTPB-GFP) iPS cells into lung organoids and showed the presence of SFTPB genetic material without protein expression at the iPSC and early endodermal phases of differentiation and the presence of both genetic and protein material in the lung organoid. We showed normal lamellar bodies on TEM and the functionality of ATII cells in the hiPro133 + SFTPB-GFP lung organoids as demonstrated by the secretion of tubular myelin and surface-active DPPC into the culture medium. Together, our data show that lentiviral correction of surfactant protein B deficiency *in vitro* is feasible but whether this is achievable *in vivo* remains to be determined.

We chose to use lentiviral infection for many reasons, including evidence for its usefulness in other pulmonary diseases. Cystic fibrosis is a monogenic lung disease that has been a target of gene therapy. *In vitro* and *in vivo* studies have suggested that only 5–10% of normal CFTR function is required to reverse the chloride channel defect^55,56^, yet nearly every cell in the airway must be corrected to reverse the excess activity of the epithelial sodium channel (ENaC)^57,58^ which is important in the reabsorption of sodium ions. Clinically, it may be more practical to have a minority of cells expressing much higher levels of the target protein to restore normal function. This would also apply to the surfactant protein B mutation. Some children with SFTPB mutations have a relatively milder phenotype^59^ and this has led to the consideration of whether a small amount of mature SFTPB (approximately 8% to 10% of control levels) is sufficient for the milder phenotype. This hypothesis is supported by studies in which mice were genetically engineered to manipulate their production of SFTPB and developed respiratory failure when their SFTPB levels fell to 20–30% of those observed in controls^60^. *In vivo* patient specific cellular transplantation of corrected ATII cells would be an ideal therapeutic option in SFTPB deficiency, but studies have shown that after a relatively efficient initial deposition of cells through the trachea, long-term uptake, retention and differentiation of transplanted cells is relatively inefficient in the absence of ongoing injury^61^. This may support genetically engineering a small number of cells to secrete higher levels of protein.

Our transgene was inserted in the hiPro133 iPS cells and expression was confirmed through the accompanying GFP signal as well as the expression of SFTPB transcripts thoughout the differentiation. We also examined protein expression of SFTPB and did not detect it during the pluripotent state or through the stages of endodermal differentiation but did detect SFTPB protein after lung organoid differentiation. This may be explained by multiple factors including the lack of translation of the SFTPB gene early on in development when the protein product is not required, the lack of activators and/or inducers to aid in translation at such an early stage or the complex processing of the SFTPB mRNA product which may only occur in a specific cell type.

Our lung organoid differentiation protocol is unique in many ways. Compared to the other mixed epithelial/mesenchymal lung organoid protocols, we don’t collect anterior foregut spheroids as the starting population for 3D organoids^9,11^ or sort for surface markers of NKX2-1 positive cells^12,32^, but wait until the lung progenitor stage to pass the monolayer cells into matrigel. We don’t use an external mesenchymal cell population to interact with the epithelial cell population^32^ and our differentiation takes only 6 weeks as opposed to many months^9^. Our differentiation protocol is also free of any animal serum (FBS) which may introduce unknown growth factors into the differentiation cocktails^11^. We have shown the presence of lung cells at different stages of lung development including progenitor branching tip cells, proximal airway cells and distal progenitor and mature alveolar lung cells. Our efficiency of approximately 22% SFTPC^+^ cells is also higher than other mixed organoids quoted in the literature (eg. 5% in Dye *et al*.^11^) but less than that of pure alveolospheres (51% in Yamamoto *et al*.^13^ and 69% in Jacob *et al*.^12^). These organoids can be used to model human lung disease such as bronchopulmonary dysplasia and viral infections since these affect multiple cell types of the lung, not just a single epithelial cell population. Interactions between mesenchyme and epithelium can also be examined since some of the supporting cells in the organoids stained positive for lung mesenchymal markers^7^ including vimentin, PDGFRA and SMA.

There was a change in the morphology of the organoids in the third week of the 3D differentiation in matrigel after the addition of DCI. Twenty four hours after exposure to DCI, some of the dense branching organoids began “ballooning” out and forming transparent spheres. The type of iPSC line determined the number of organoids that became round and transparent. The hiPro133 (SPB deficient) derived organoids all became fully transparent spheres, the hiPro133 + SFTPB-GFP derived organoids contained 75% transparent spheres and the wt heCA1 cells contained 50% transparent spheres at day 40 with the rest remaining dense branching organoids. We believe this morphological change is due to the maturation of some specific cell types in response to DCI but have not elucidated which types of cell are affected. This cocktail of small molecules and hormones may be targeting the epithelial and/or the mesenchymal cell populations and activating a signaling pathway which causes the morphological changes. We aim to explore this phenomenon with bulk RNA seq after separating out the opaque and transparent organoids to determine the gene expression changes that occur after the introduction of DCI.

Our organoids did not express CD31, a well-known marker for endothelial cells (data not shown), suggesting that we did not have a vascularized organ.

Another interesting phenomenon we noted in our differentiation is the presence of progenitor branching tip cells in organoids at the same point in time of the differentiation as distal alveolar organoids. During the pseudoglandular stage, distal epithelial progenitors at the tips co-express SOX2 and SOX9^48^. This double-positive population is no longer present by the canalicular stages of development^62^ and instead SOX9 becomes isolated to the distal alveolar population^63^. These two different populations of organoids in a single transwell may indicate that upon passaging the LPC cells into 3D matrigel, organoids may not uniformly differentiate. By passaging the LPCs as cell aggregates, we may be creating organoids at various stages of lung development in a single transwell. This may be due to the LPCs containing a variety of SOX2^+^ and SOX9^+^ cells, either singly expressed or co-expressed, representing different cell types entering the early phases of lung development. To confirm this phenomenon, we would need to assess the transcriptomes of the LPCs as well as of the developing organoids and compare their gene expression patterns at each stage of lung development.

Finally, despite the importance of SFTPB in distal alveolar functions such as surfactant stability and SFTPC post translational modification, SFTPB-deficient iPSC derived organoids also showed changes in proximal airway gene and protein expression. SFTPB is expressed in Club cells in its pro-peptide form and may affect proximal airway cell differentiation if absent. Post translational modification of SFTPB has an important role in maintaining lung homeostasis in the mouse lung, yielding two different protein products, SP-B^M^ and SP-B^N^, which are involved in lowering surface tension and promoting host defense, respectively^64^. Studies have shown that the balance of these two pro-peptides may alter susceptibility to lung infections^65^ but how they impact lung development is unknown.

Taken together our work shows that human lung development and disease can be studied *in vitro* to discover new mechanisms of disease, patient specific therapeutic options and be the first step towards a cure for human lung disease.

## Methods

### Derivation of hiPS cells from fibroblasts

Fibroblasts from a patient with the p.Pro133GlnfsTer95 SFTPB deficiency (generously donated by Drs. Cole and Hamvas, Washington University, St. Louis and Northwestern University, Chicago) were given to the Center for Commercialization of Regenerative Medicine (CCRM, Toronto) for hiPS cell derivation as well as fibroblasts from an individual with a normal surfactant profile. Reprogramming factors hOct4, hSox2, hKlf4 and hMyc (CytoTune iPS Sendai Reprogramming Kit) were added to the fibroblasts and incubated for 24 hours. After 24 hours, the viral solution was removed and replaced with fresh fibroblast expansion medium and changed daily for a week. After day 7, cells were re-seeded onto 10-cm tissue culture dishes coated with human foreskin fibroblasts. Human ESC complete media made up of DMEM/F12, 20% (v/v) knockout serum replacement (Gibco (Life Technologies)), 0.1 mM β-mercaptoethanol (Sigma-Aldrich) and 10 ng/ml FGF2 (R&D) was exchanged daily until approximately day 28, or until colonies were ready for picking. Authentic colonies were identified, cleaned and expanded on human foreskin fibroblast-coated dishes in hESC complete media. The iPS cells were characterized through karyotyping, immunostaining and FACS for OCT4, NANOG, SSEA4, TRA160, TRA181, and RT-PCR for pluripotency markers and cell line authentication (STR analysis) to confirm parental origin.

### Sfu1 digestion

We performed PCR amplification in 10-μL reaction volumes in each well of a 96-well plate as previously described^26^: Five μL of supernatant containing DNA template; 1.6 μL deoxynucleotides (final concentration 200 μM); 0.5 μL of 5% DMSO; 0.75 U (0.1 μL) *Taq* polymerase (Sigma Chemical Co., St. Louis, MO, U.S.A.), and 2 μL of Buffer J [300 mM Tris HCl, 75 mM (NH_4_)_2_SO_4_, and 10 mM MgCl_2_, Invitrogen, Carlsbad, CA, USA]. To increase the specificity of amplification of the target sequence, we used 36-monomer primers (sense: 5′ TAA CTC CTT GGC ACT CGT GAA CTC CAG CAC CCT G 3′; antisense: 5′ GCT GGC TGG GGT GCT GTG TGT GTG GCT CCC CCA TG 3′) to amplify a 354-bp fragment of the SFTPB gene (nucleotides 1320–1674) that contains the 121ins2 mutation. We amplified extracted template in a thermocycler (MJ Research, Watertown, MA, U.S.A.) as follows: 3.5 min at 95 °C for initial denaturation, 30 cycles denaturation at 95 °C for 30 s each, annealing at 68 °C for 45 s, elongation at 72 °C for 80 s, and a final elongation at 72 °C for 3 min. We performed restriction enzyme digestion with *Sfu* I as previously described^16^. Briefly, we digested amplicons in each well with 2.5 U (5 μL) *Sfu* I in H buffer (Roche Molecular Biochemicals, Indianapolis, IN, USA) by incubating at 37 °C for 90 min. We added 5 μL of loading buffer and subjected each digest to metaphor/agarose-ethidium bromide gel electrophoresis. Each gel was photographed under UV light and scanned into a database.

### Lentiviral infection of human iPS cells with SFTPB_wt_-GFP insert

GeneCopoeia’s EX-M0587-Lv201 lenti-vector was used (M0587). The vector contained a CMV promoter driving the open reading frame (ORF) of homo sapiens surfactant protein B (Accession: BC032785.1) followed by SV40-eGFP-IRES-puromycin. To generate the lentivirus, the HIV-based EX-M0587-Lv201 lenti-vector, in conjunction with GeneCopoeia’s Lenti-Pac™ HIV Expression Packaging vectors were co-transfected into HEK293T cells using GeneCopoeia’s EndoFectin™ Lenti Transfection Reagent. Cells were incubated in the presence of 5% CO_2_ at 37 °C overnight. Growth medium was changed to Opti-MEM containing 3% (v/v) FBS with the addition of GeneCopoeia’s Titerboost. Conditioned medium was collected after 24, 48 and 72 hours of incubation and centrifuged at 2,000 × g for 30 minutes. The supernatant was transferred to a new tube and PEG 6000 solution was then added to make the final PEG 6000 concentration to be 8.5% (w/v) and the final NaCl concentration to be 0.3 M. The mixture was incubated on ice for 3 to 6 hours and then centrifuged at 2,000 × g for 30 minutes. The viral particle containing pellet was resuspended by pipetting in 1/20 of the original harvest volume of Opti-MEM. Infection of the lentivirus was performed on 24-well or 6-well plates. Wt iPS and Pro133 iPS cells were plated one day before infection at 60–70% confluence. Five million transduction units (TU) per milliliter of infectious particle was used to infect 10^6^ cells together with polybrene (final concentration 80 µg per milliliter). Growth medium was changed one day after infection, with the addition of puromycin (0.25 µg per milliliter). Cells were selected under puromycin for more than two weeks and positive cells were verified by GFP expression in iPS cells using fluorescence microscopy. For the verification of SFTPB expression *in vitro*, total RNA was extracted from iPS cells using the RNeasy Mini Kit (Qiagen). Purified total RNA was reverse transcribed to cDNA using the miRNA reverse transcription kit with SFTPB specific primers (Life Technologies). qRT-PCR was performed and analyzed with StepOne Software (Applied Biosystems). For sequences of primers, see Supplementary Table S1.

### Maintenance of hES and iPS cells

Sendai generated human dermal fibroblasts iPSC lines (hi31616, hiPro133 and hiPro133 + SFTPB-GFP cells) were cultured on matrigel coated plates in a MTESR 1 medium (STEMCELL Tech 85850). Medium was changed daily, and cells were passaged using ReLeSR (STEMCELL Tech 05872) every 5–7 days. Cultures were maintained in an undifferentiated state in a 5% CO_2_/air environment. Human iPSC differentiations were carried out in a 5% CO_2_/21% O_2_/90% N_2_ environment.

### Directed differentiation of iPS cells to lung progenitor cells

When the human iPSCs reached 70% confluence (Day 0), the cells were incubated in 10 μM of Y-27632 (Wako) for one hour and then in accutase (Innovative Cell Technologies) for 20 minutes at 37 °C^32^. The detached iPSCs were then dissociated into single cells via pipetting, and then seeded on matrigel-coated plates (BD Biosciences) at a density of 1.75 × 10^5^ cells/cm^2^ in DE induction medium made up of RPMI1640 (Life Technologies), 1x B27 supplement, 1% HEPES, 1% glutamax and 50 U/ml of penicillin/streptomycin. This basal medium was supplemented with 100 ng/ml of human activin A (R&D systems), 1 μM of CHIR99021 (Stemgent), and 10 μM of Y-27632 On days 2–4, only activin was added. On Day 5, the medium was changed to AFE induction medium. Depending on cell line and confluence, some DE wells were passed in ratios of 1:1–1:3, while others underwent AFE induction without passage. AFE and LPC induction basal mediums consisted of 3:1 IMDM:F12 (Life Technologies), 1x B27 and N2 supplements (Life Technologies), 50 U/ml of penicillin/streptomycin, 0.25% BSA, 0.05 mg/ml of L-ascorbic acid (Sigma-Aldrich), and 0.4 mM of monothioglycerol (Wako)^30^. This medium was supplemented with 10 μM SB431542 (R&D Systems) and 2 μM Dorsomorphin (Stemgent) for AFE induction for 3 days.

On Day 8, the medium was changed to LPC induction medium, containing the basal medium with 10 ng/ml of human recombinant BMP4 (R&D Systems), 0.1 μM of all-trans retinoic acid (RA) (Sigma-Aldrich) and 3 μM of CHIR99021. Media was changed every 2 days for 8–11 days.

### 3D culture of LPC cells to lung organoids

The protocol for the 3D culture was modified from previous reports^12,32^. Monolayer LPC cells were incubated in 10 μM of Y-27632 for one hour then placed in accutase for 10 min. 10–40 × 10^4^ cells were resuspended in 200 μl of undiluted growth factor reduced matrigel and seeded onto a 12-well cell culture insert (BD Biosciences). One ml of 3D step 1 media (with FGF7 (10 ng/m), FGF10 (10 ng/ml), CHIR (3 um) and EGF (10 ng/ml) was added to the lower chamber and changed every other day for 8 days. D basal media was identical to the AFE/LPC induction basal media. On day 25, the media was changed to 3D step 2 medium consisting of FGF7 (10 ng/ml), FGF10 (10 ng/ml), CHIR (3 μM), RA (0.1 μM), EGF (10 ng/ml) and VEGF/PIGF (10 ng/ml). Media was changed every 2 days for 8 days. Finally, 3D step 3 media was added which was the same as step 2 with the addition of Dexamethasone (50 nM), cAMP (100 μM) and IBMX (100 μM). Media was changed every 2 days for 7 days.

### Flow cytometry

The cells grown in 3D matrigel were dissociated with dispase (17105041 ThermoFisher) at 37 °C for 1 hour centrifuged at 300 g × 5 min and washed in 1x cold PBS. To generate single cell suspensions, cell pellets were incubated in TrypLE for 10–15 minutes. The reaction was diluted in DMEM/F12 (Life Technologies) with 2% (v/v) FBS and centrifuged at 300 g at room temperature. The cell pellets were immersed with sorting buffer 3% (v/v) FBS in PBS, cell aggregates were removed using a cell strainer with a 70-µm pore size (BD falcon) and single cells were collected. For surface antigens, the cells were incubated with primary antibodies for 30 minutes with gentle shaking, washed twice with 3% (v/v) FBS/PBS, and if necessary, incubated with the secondary antibodies for 30 minutes followed by two more rinses with 3% (v/v) FBS/PBS. For intracellular antigens, the Flow Cytometry Fixation & Permeabilization Buffer Kit I, Flow Cytometry Permeabilization/Wash Buffer I and Flow Cytometry Staining Buffer kits (R&D). The cells were analyzed using a BD FACS Aria II flow cytometer (BD Biosciences). Unstained controls were used for gating conjugated antibodies and secondary only controls were used for gating unconjugated antibodies. The list of antibodies used for FACS is shown in Supplementary Table S2.

### Immunofluorescence staining

The cells grown on cover slips (2D culture) were fixed with 4% (v/v) paraformaldehyde (PFA) in PBS (R&D Systems) for 15 minutes at RT. After washing three times with PBS, the cells were immersed in 0.5% (v/v) Triton X-100 in PBS for 10 minutes at RT, followed by incubation with blocking solution consisting of 5% (v/v) normal donkey serum (Millipore), 3% (w/v) BSA (Sigma-Aldrich) and 0.5% (v/v) Triton X-100 in PBS for 60 minutes at RT. The cells were then incubated in the primary antibody solution for 60–120 minutes at RT or overnight at 4 degrees Celsius, followed by washing three times with 0.1% (v/v) tween in HBSS. The cells were incubated in the secondary antibody solution for 60 minutes at RT and washed three times with 0.1% (v/v) tween in HBSS. All primary and secondary antibodies used in the present study were diluted in blocking solution as indicated in Supplementary Table S2. Nuclei were counterstained with Hoechst-33342 or DAPI (Thermo Fisher) at 1:1,000 for 5 minutes. Controls were stained with secondary antibody only. For the 3D cultures, after fixation with PFA for one hour, the 3D matrigel disc in the transwell containing the organoids was embedded in paraffin, sectioned at 5 μm thickness, dewaxed, rehydrated then stained with hematoxylin and eosin (H&E) to visualize the spheroids under a light microscope. For immunofluorescence staining of the 3D cultures, antigen retrieval on the fixed sections was performed with 10 mM sodium citrate, pH 6.0. The sections were then blocked and stained with antibodies as described above. Images were collected on a Leica confocal SPE microscope.

### Western blotting

Cells were harvested by washing cell three times with PBS (Cellgro, 21-031-CV), dislodged using cell scrapers into 1x RIPA buffer (Thermo, 89901) with 1x protease and phosphatase inhibitor cocktail (“Halt” Thermo Scientific Cat, 1861280). Samples were subjected to BCA assay (Pierce, 23228, 23224) for protein quantitation. 25 ul of 4x LDS loading dye (Life Tech, NP0007) and 10 ul of DTT (Acros, 32719) are added to 65 ul of sample. Typically, 6–10 ug of protein were loaded per well of a 4–12% Bolt gradient gel (Life Tech, NW04127BOX). Gels were run at 160 V for 70 minutes and transferred at 30 V for 80 minutes using the BOLT Western Blotting system by Life Technologies (Running Buffer: NP0002; Transfer Buffer: NP0006-01). Blots were blocked in TBS (Tirzma Acid (Sigma, T3253) plus Trizma base (Fisher, BP152-1), with 5% milk (Apex, 20–241)) for 1 hour or overnight at 4 C. Blots were then incubated with primary antibody at 1:500 in TBST (TBS with Tween 20 (Acros, 23336–2500)) and 5% BSA (Fisher, BP1600-100) either overnight at 4 °C or room temperature for 2 hours. Antibodies used: Rabbit anti-SPB (7 Hills, 48604) and Rabbit anti-SPC (LS Biosciences, LS-B9161). Blots were then washed with TBST and incubated with appropriate secondaries: Goat anti-Rabbit-680 (Life Tech, A21109), Goat anti-Mouse-800 (Li-Cor, 926-32210), at a 1:4,000 dilution. Blots were then washed with TBST three times for 10 mins and then imaged on the Li-Cor Odyssey Imager. Lanes were quantified using the Li-Cor Image Studio software version 2.0.

### RNA isolation, cDNA preparation and real-time PCR

Total RNA was isolated using the Arcturus PicoPure RNA isolation Kit (Invitrogen) according to the manufacturer’s manual. First-strand cDNA was synthesized from 80 ng of total RNA using the SuperScript IV First-Strand Synthesis System (Invitrogen). The cDNA samples were amplified using 2X SYBRSelect Master Mix (Invitrogen) with ABI7300 Real-Time PCR System (Life Technologies). All reactions were started at a cycle of 50 °C for 2 minutes, 95 °C for 10 minutes, followed by 40 cycles of 95 °C for 15 seconds, 60 °C for 1 minute. The PCR reactions were performed in triplicate for each sample. The level of expression of each gene was calibrated to that of the housekeeping gene, β-actin (ACTB), and compared to the level of the expression of each gene in the fetal human lungs. All primer sets are shown in Supplementary Table S1.

### Electron microscopy

Samples were fixed in a solution containing 4% (v/v) formaldehyde and 1% (v/v) glutaraldehyde in 0.1 M phosphate buffer (pH 7.4) and then post fixed in 1% (w/v) osmium tetroxide. The specimens were then dehydrated in a graded series of acetone from 50% to 100% and subsequently infiltrated and embedded in Epon-Araldite epoxy resin. Ultrathin sections were cut with a diamond knife on the Reichert Ultracut E (Leica Inc). Sections were stained with uranyl acetate (2–3%) and lead citrate (0.1–0.4%) before being examined in a JEM-1011 (JEOL USA Corp) microscope.

### Measurement of phosphatidylcholines

Medium (500 µL) conditioned by the of organoids was transferred to siliconized glass tubes containing 500 µL ultra-pure water and spiked with an internal standard (1 mg PC-17:0/17:0). After addition of 2 mL of methanol/chloroform (1:1) samples were vortexed, centrifuged and the chloroform layer collected. After drying, samples were reconstituted in 100 µL ethanol that was acidified with 0.2 mL of formic acid and transferred to siliconized mini vials for analysis. Liquid chromatography tandem mass spectrometry was performed on an Agilent 1290 Series binary pump (Agilent Technologies Inc., Santa Clara, CA, USA) coupled to an API5500 triple-quadruple mass spectrometer (SCIEX, Concord, ON, Canada). Prior to analysis, Multiple Reaction Monitoring (MRM) mass transition parameters were optimized by infusion of pure PC standards (5 µl/min of 1 ug/mL). MS analysis was performed in positive electrospray ionization mode. For quantitative analysis a separate standard curve was generated for each analyte measured using MRM area ratios (Analyte Peak Area/IS Peak Area). Results were then calculated by plotting the sample area ratios against their respective analyte standard curve.

### Statistical analysis

Statistical analysis was performed using Student’s t-test or 2-way Anova for gene expression and Western blot pixel analysis (SPSS 12.0 software; SPSS) with significance set at P < 0.05. All data are expressed as mean ± standard error of the mean (s.e.m.) of three or more experimental replicates. For immunofluorescence efficiency calculation, cells were defined by the presence of a nucleus (DAPI) in tissue sections. Cell marker positive cells were calculated as a percentage of total DAPI cells counted per field and analyzed by Student’s t test using SPSS 12.0 software. Full organoids were counted at 10x magnification to determine efficiency of specific protein expression. We performed 3 technical and at least 3 biological repeats of organoids from each cell line and experiment.

## Supplementary information

Supplementary Figures

Supplementary Tables

## Data Availability

The authors declare that the data supporting the findings of this study are available within the paper and its Supplementary Information Files.
