# Supplementary material for: Reversal of Surfactant Protein B Deficiency in Patient Specific Human Induced Pluripotent Stem Cell Derived Lung Organoids by Gene Therapy
Source: Sci Rep. 2019 Sep 17;9:13450. doi: 10.1038/s41598-019-49696-8 (PMC6748939; doi:10.1038/s41598-019-49696-8)
Supplement: Supplementary file 1 — Supplementary Figures [file 41598_2019_49696_MOESM1_ESM.pdf]

Supplementary Figures

Reversal of Surfactant B Deficiency in Patient Specific Human Induced Pluripotent Stem Cell Derived Lung Organoids by Gene Therapy

Sandra Lawrynowicz Leibel*[1,2,3], Alicia Winquist[2,3], Irene Tseu[4], Jinxia Wang[4], Daochun Luo[4], Sharareh Shojaie[4], Neal Nathan[2,3], Evan Snyder[1,2,3], Martin Post[4,5]

Affiliations

1. Department of Pediatrics, University of California, San Diego, Rady Children's Hospital, San Diego, La Jolla, CA, USA.
2. Sanford Burnham Prebys Medical Discovery Institute, La Jolla, CA, USA
3. Sanford Consortium for Regenerative Medicine, La Jolla, CA, USA.
4. Translational Medicine Program, Peter Gilgan Centre for Research and Learning, Hospital for Sick Children, Toronto, Ontario, Canada.
5. Department of Physiology, University of Toronto, Toronto, Ontario, Canada

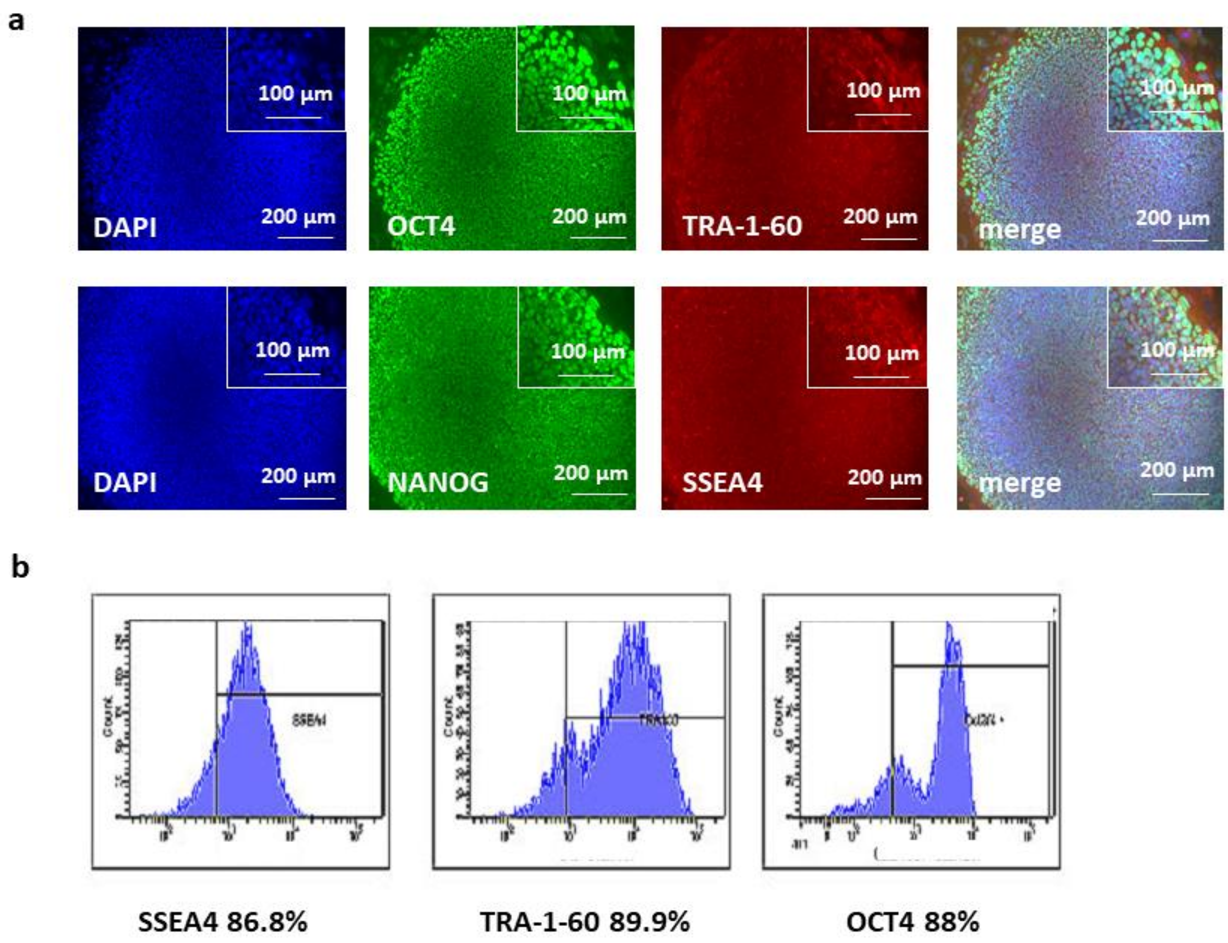

Supplementary Figure S1: Human iPS cells reprogrammed from Pro133 dermal fibroblasts express pluripotency markers. (a) Positive immunofluorescence pluripotency markers for OCT4, TRA1-60, NANOG and SSEA4. (**b**) Flow cytometry for pluripotency markers SSEA4, TRA-1-60 and OCT4.

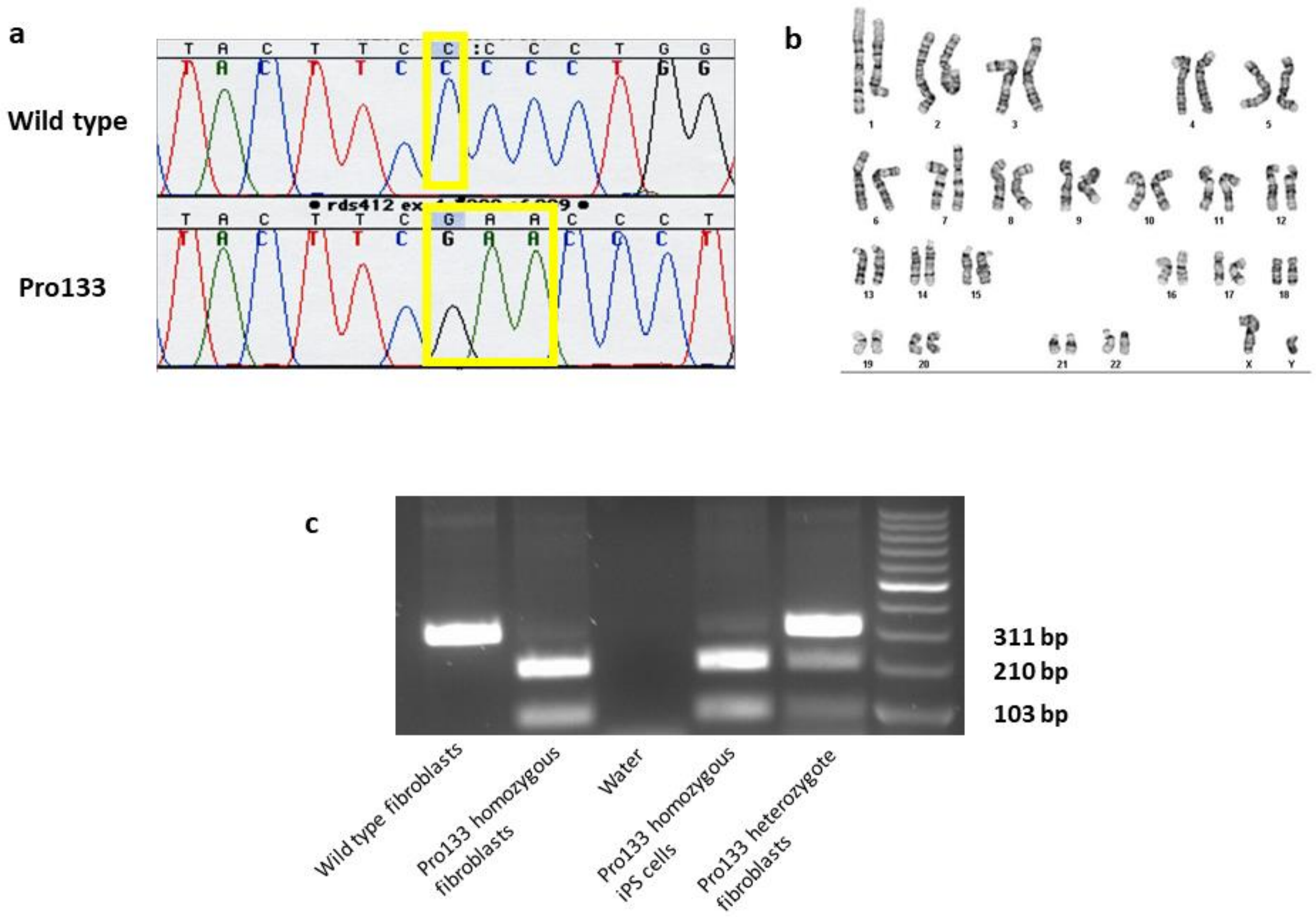

Supplementary Figure S2: Human iPS cells derived from p.Pro133GlnfsTer95 (Pro133) dermal fibroblasts express the SFTPB mutation. (**a**) Sequencing of wild-type and hiPro133 iPS cells. Yellow box indicates normal sequence in the wild type box above and mutant Pro133 sequence in the box below. (b) Karyotype of Pro133 iPS cells. (**c**) SfuI digestion of normal, heterozygous and homozygous hiPro133 fibroblasts and hiPS cells. The homozygous mutation reveals two distinct bands (210 and 103 bp) in the SFTPB deficient iPSCs and fibroblasts, one band (311bp) in the wild type fibroblasts and three bands in the heterozygous fibroblasts.

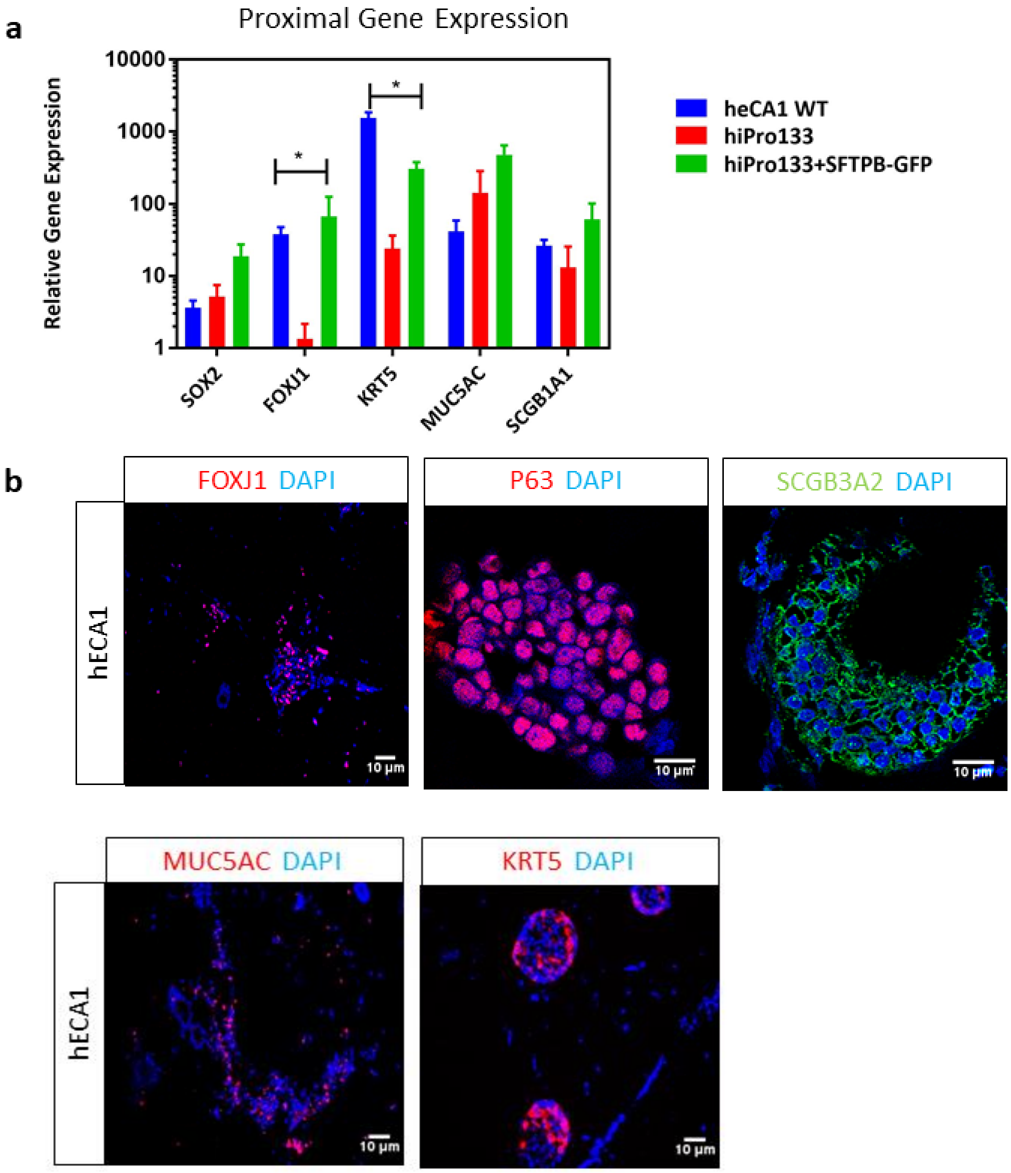

Supplementary Figure S3: Expression of proximal lung cell markers in lung organoids derived from heCA1 wt cells. (**a**) Gene expression of proximal epithelial lung cells present in day 40 lung organoids. * indicates significant differences with p-value <0.05 (n=3-5 separate differentiations). (**b**) Immunofluroescence of proximal epithelial lung cell markers FOXJ1, P63, SCGB3A2, MUC5AC and KRT5 in day 40 lung organoids. Data are representative of > 15 independent experiments. (Scale bars = 10 μM)
