# Supplementary material for: Reversal of Surfactant Protein B Deficiency in Patient Specific Human Induced Pluripotent Stem Cell Derived Lung Organoids by Gene Therapy
Source: Sci Rep. 2019 Sep 17;9:13450. doi: 10.1038/s41598-019-49696-8 (PMC6748939; doi:10.1038/s41598-019-49696-8)
Supplement: Supplementary file 2 — Supplementary Tables [file 41598_2019_49696_MOESM2_ESM.pdf]

Supplementary Tables

Reversal of Surfactant B Deficiency in Patient Specific Human Induced Pluripotent Stem Cell Derived Lung Organoids by Gene Therapy

Sandra Lawrynowicz Leibel*[1,2,3], Alicia Winquist[2,3], Irene Tseu[4], Jinxia Wang[4], Daochun Luo[4], Sharareh Shojaie[4], Neal Nathan[2,3], Evan Snyder[1,2,3], Martin Post[4,5]

Affiliations

1. Department of Pediatrics, University of California, San Diego, Rady Children's Hospital, San Diego, La Jolla, CA, USA.
2. Sanford Burnham Prebys Medical Discovery Institute, La Jolla, CA, USA
3. Sanford Consortium for Regenerative Medicine, La Jolla, CA, USA.
4. Translational Medicine Program, Peter Gilgan Centre for Research and Learning, Hospital for Sick Children, Toronto, Ontario, Canada.
5. Department of Physiology, University of Toronto, Toronto, Ontario, Canada

Table S1: Primer sets used in RT-PCR

Gene	Forward primer (5’	Reverse primer (5’
**KRT5**	GGAGTTGGACCAGTCAACATC	TGGAGTAGTAGCTTCCACTGC
**MUC5AC**	CCATTGCTATTATGCCCTGTGT	TGGTGGACGGACAGTCACT
**FOXJ1**	GAGCGGCGCTTTCAAGAAG	GGCCTCGGTATTCACCGTC
**SFTPC**	CACCTGAAACGCCTTCTTATCG	TGGCTCATGTGGAGACCCAT
**SCGB1A1**	TTCAGCGTGTCATCGAAACCCT	ACAGTGAGCTTTGGGCTATTTTT
**FOXA2**	AGGAGGAAAACGGGAAAGAA	CAACAACAGCAATGGAGGAG
**SOX17**	AAGGGCGAGTCCCGTATC	TTGTAGTTGGGGTGGTCCTG
**TG**	AGAAGAGCCTGTCGCTGAAA	TTGGACCAGAAGGAGCAGTC
**NKX6.1**	ATTCGTTGGGGATGACAGAG	CGAGTCCTGCTTCTTCTTGG
**AFP**	TGGGACCCGAACTTTCCA	GGCCACATCCAGGACTAGTTTC
**CFTR**	CTATGACCCGGATAACAAGGAGG	CAAAAATGGCTGGGTGTAGGA
**NANOG**	TGATTTGTGGGCCTGAAGAAA	GAGGCATCTCAGCAGAAGACA
**CDX2**	CTGGAGCTGGAGAAGGAGTTTC	ATTTTAACCTGCCTCTCAGAGAGC
**SOX2**	GCACATGAAGGAGCACCCGGATTA	CGGGCAGCGTGTACTTATCCTTCTT
**FOXG1**	CTCCGTCAACCTGCTCGCGG	CTGGCGCTCATGGACGTGCT
**SOX9**	GAGGAAGTCGGTGAAGAACG	ATCGAAGGTCTCGATGTTGG
**PAX6**	TCTTTGCTTGGGAAATCCG	CTGCCCGTTCAACATCCTTAG
**OTX2**	GTGGGCTACCCGGCCACCC	GCACCCTCGACTCGGGCAAG
**Pdpn**	GTCCACGCGCAAGAACAAAG	GGTCACTGTTGACAAACCATCT
**P2X7**	TATGAGACGAACAAAGTCACTCG	GCAAAGCAAACGTAGGAAAAGAT
**DLX3**	CTCGCCCAAGTCGGAATATAC	CTGGTAGCTGGAGTAGATCGT
**Brachyury**	CAGTGGCAGTCTCAGGTTAAGAAGGA	CGCTACTGCAGGTGTGAGCAA
**NKX2.5**	CCCAGCCAAGGACCCTAGA	GCGTTGTCCGCCTCTGTCT
**ABCA3**	GCCCTCTTTACACTCAGTTTTCA	GACGAGCAGTTGTCGTACCTAAT
**SFTPB**	TCTGAGTGCACCTCTGCATGT	TGGAGCATTGCCTGTGGTATGG
**ACTB**	TTTGAATGATGAGCCTTCGTGCCC	GGTCTCAAGTCAGTGTACAGGTAAGC
**AQP5**	GCCATCCTTTACTTCTACCTGCTC	GCTCATACGTGCCTTTGATGATGG
**LAMP3**	ACTTCAACATCGACCCCAAC	CACTCACGCACTTGAAGGAA
**P1407S**	GAACTCCAGCACCCTGGGGGA	GCTCCCCATGGGTGGGCACA

Gene	Ref number
**NKX2-1**	QT00015981
**P63**	HP102339

Table S2: Antibodies used in FACS and Immunofluorescence. W=western blot; F=FACS

Primary Antibodies	Company	Catalogue #	Dilution rate
Embryonic Stem Cell Marker Panel	abcam	ab109884	1:300
SSEA4-AF 488	Biolegend	330412	5ul/1x10^6 cells (F)
TRA-1-60-PE	Biolegend	330610	5ul/1x10^6 cells (F)
OCT4-647	Biolegend	653710	5ul/1x10^6 cells (F)
CXCR4-PE	R&D Systems	FAB170P	1:200 (F)
EPCAM-488	BD Bioscience	347197	1:500 (F)
FOXJ1	Invitrogen	14-9965-82	1:300
GFP	R&D Systems	MAB42401R	1:1000
HOPX	Santa Cruz Biotech	sc-398703	1:200
HTII-280	Terrace Biotech	TB-27AHT2-280	1:150
ID2	abcam	ab52093	1:300
KRT5	abcam	ab52635	1:200
MUC5AC	Millipore	MAB2011	1:200
NKX2-1	abcam	ab76013	1:300
NKX2-1-APC	LS-BIO	LS-C264437	1:1000 (F)
p63	Boster	PB9152	1:250
PDGFRA	Cell Signalling	3169S	1:300
PDPN	abcam	ab10288	1:500
pSPC	abcam	ab40871	1:250
SCGB3A2	abcam	ab181853	1:300
SOX2	Invitrogen	MA1-014	1:200
SOX9	R&D Systems	AF3075	1:200
SPB	abcam	ab40876	1:250
SPB (mature)	7 Hills	48604	1: 1500 (F) 1:500 (W)
SPC (mature)	LS Bio	LS-B9161	1:100 (F); 1:500 (W)
Vimentin-PE	Santa Cruz Biotech	sc-6260	1:100
ZO1	Invitrogen	33-9100	1:250

Secondary Antibodies	Company	Catalogue #	Dilution rate
Donkey anti-mouse IgG (Alexa 488)	Abcam	Ab150105	1:500
Donkey anti-mouse IgG (Alexa 555)	Abcam	Ab150106	1:500
Donkey anti-mouse IgG (Alexa 647)	Abcam	Ab150107	1:500
Donkey anti-rabbit IgG (Alexa 488)	Abcam	Ab150073	1:500
Donkey anti-rabbit IgG (Alexa 555)	Abcam	Ab150074	1:500
Donkey anti-rabbit IgG (Alexa 647)	Abcam	Ab150075	1:500
Donkey anti-goat IgG (Alexa 488)	Abcam	Ab150129	1:500
Donkey anti-goat IgG (Alexa 555)	Abcam	Ab150130	1:500
Donkey anti-goat IgG (Alexa 647)	Abcam	Ab150131	1:500
